# Openness and Transparency Promotion With Existing Longitudinal Data: A Worked Example of a Coordinated Analysis

**DOI:** 10.1093/geroni/igaa057.1878

**Published:** 2020-12-16

**Authors:** Daniel Mroczek, Eileen Graham, Emily Willroth

**Affiliations:** 1 Northwestern University, Chicago, Illinois, United States; 2 Northwestern University Feinberg School of Medicine, Evanston, Illinois, United States

## Abstract

The application of openness and transparency principles is challenging when using existing or ongoing long-term longitudinal data. One technique that promotes replicability and also is consistent with openness and transparency principles is coordinated analysis. Such analyses, especially when done with a large number of extant longitudinal datasets, tend to draw upon values of data sharing, revelation of code and scripts, and pre-registration. Thus coordinated analyses often provide good examples of how multiple transparency and openness values can come together. We will demonstrate this by presenting two recent large-scale coordinated analyses. One was a 15-study investigation of personality and mortality risk (Graham et al., 2017). The second is a new 16-study investigation of personality trajectories (Graham et al., under revision). We show how multi-study designs are congruent with open science and transparency ideas in the context of longitudinal and other secondary data.

